# Using evolved gas analysis – mass spectrometry to characterize adsorption on a nanoparticle surface†

**DOI:** 10.1039/c9na00098d

**Published:** 2019-06-04

**Authors:** Jordi Martínez-Esaín, Teresa Puig, Xavier Obradors, Josep Ros, Jordi Farjas, Pere Roura-Grabulosa, Jordi Faraudo, Ramón Yáñez, Susagna Ricart

**Affiliations:** Departament de Química, Universitat Autònoma de Barcelona 08193 Bellaterra Spain; Institut de Ciència de Materials de Barcelona (ICMAB-CSIC) 08193 Bellaterra Spain ricart@icmab.es; University of Girona Campus Montilivi, Edif. PII E17071 Girona Spain

## Abstract

The surface chemistry of nanoparticles is the key factor to control and predict their interactions with molecules, ions, other particles, other materials, or substrates, determining key properties such as nanoparticle stability or biocompatibility. In consequence, the development of new techniques or modification of classical techniques to characterize nanoparticle surfaces is of utmost importance. Here, a classical analysis technique, thermally evolved gas analysis – mass spectrometry (EGA-MS), is employed to obtain an image of the nanoparticle–solvent interface, unraveling the molecules present on the surface. As the use of complementary techniques is urged, the validity of EGA-MS characterization is corroborated by comparison with a previously reported surface characterization method. Previous studies were based on several experimental techniques and MD simulations using YF_3_ nano/supraparticles and LaF_3_ nanoparticles as model systems. We demonstrate the applicability of this technique in two differently sized systems and two systems composed of the same ions on their surface but with a different inorganic core (*e.g.* LaF_3_ and YF_3_ nanoparticles). The results described in this paper agree well with our previous results combining experimental techniques and MD simulations. EGA-MS not only revealed the ions attached to the nanoparticle surface but also shed light on their coordination (*e.g.* citrate attached to one or two carboxylate moieties). Thus, we show that EGA-MS is a useful and efficient technique to characterize the surface chemistry of nanoparticles and to control and predict their final properties.

## Abstract 2

EGA-MS is presented as an experimental technique to characterize the surface of LnF_3_ nanoparticles as a complementary approach to surface chemistry.

## Graphical abstract

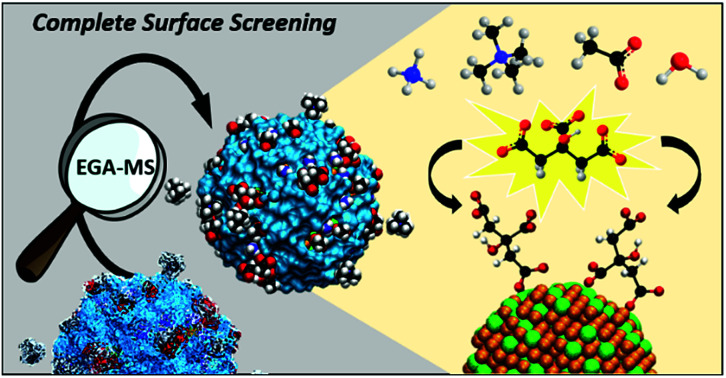

## Introduction

Nanoscale systems are formed basically from a core and a protective shell that stabilizes the system kinetically preventing its coagulation.^1^ Nowadays, the complete characterization of the nanoparticle (NP) surface is performed with the combination of several techniques to understand the complete system image.^2^ Surface chemistry has arisen as a new multidisciplinary approach to unravel and study the surface-to-ligand interactions in nanoscale systems.^3,4^ The complete knowledge of the NP surface composition is currently a complex discipline in nanoscience, due to the intricacy of developed synthetic methodologies. Currently, nuclear magnetic resonance (NMR) is a crucial technique to characterize the surface chemistry of as-synthesized NPs or those after carrying out ligand exchange.^5–8^ In addition, 2D NMR techniques (*i.e.* two dimensional nuclear Overhauser effect (NOESY) experiment)^1,4^ and saturation-transfer difference (STD)-NMR^9^ are useful approaches for the complete characterization of NP surface chemistry. However, these common techniques have drawbacks such as the fast H-D exchange and re-dispensability problems in NMR, or the overlapping of different ligands containing the same functional group in infrared spectroscopy (IR), among others. Given these limitations and difficulties in characterizing nanoscale interfaces, it is of great interest to add additional techniques to the nanoscale characterization portfolio. Our objective here is precisely to add a technique to this list, showing how evolved gas analysis – mass spectrometry (EGA-MS) can be used to characterize nanoscale surfaces.

EGA-MS is a key technique for the identification of species when a substance is subjected to a controlled temperature program. Among the vast number of applications (periodically reviewed by Materazzi *et al.*^10^ since 2006), pyrolysis and thermal stability studies on organic materials,^11,12^ and metallorganic^13^ and inorganic salts^14,15^ are the most usual. EGA is also used to identify adsorbed gases on polymers,^16^ the surface structure of catalysts,^17^ the functionalization of nanoparticles^18,19^ and carbon nanotubes to prepare graphene nanoribbons.^20^ In a vacuum, EGA-MS offers very high sensitivity and reduces the chance of secondary reactions between volatiles. Consequently, it is very suitable to analyze decomposition processes where several radicals evolve from the surface of NPs. EGA-MS allows us to monitor the evolution of the fragmentation pattern of the detected volatiles as a function of the sample temperature. The use of the fragmentation pattern of the molecules attached onto the NP surface ensures not only the detection of the presence of capping agents (as common in reported studies), but also extraction of crucial information about their behavior on the surface (*e.g.* coordination to the NP surface).

EGA-MS of NPs has been used to determine the organic amount on the surface or how strongly the stabilizer is adsorbed. Celik *et al.*^18^ reported the use of this technique coupled to a thermobalance (TG) to simultaneously measure the evolution of the NP mass. Their main objective was to detect how the stabilizer is adsorbed onto the NP surface. Finally, they postulated three different layers in the shell of the NP. On the other hand, the work reported by Slostowski *et al.*^19^ is based on the surface characterization of CeO_2_ NPs by EGA-MS and IR to unravel the presence of the alcohol used in the synthesis on the NP surface. In this case, IR was used as the key technique to detect the formation of carboxylates that bind to the NP surface with different coordinative mechanisms. In view of all this evidence, we aimed to analyze deeply different nanoscale systems using EGA-MS in a vacuum as the main technique to study the surface chemistry.

In this work, we present a simple and effective way to characterize the surface chemistry of NPs containing several ions adsorbed on the surface *via* the EGA-MS technique. We have applied the method to three different systems reported previously: (i) YF_3_ nanoparticles of ∼5 nm, (ii) YF_3_ supraparticles of ∼80 nm and (iii) LaF_3_ nanoplatelets of ∼7 nm size.^21,22^ The use of (i) and (ii) allows us to compare compounds of different sizes while maintaining the composition. Group (iii) allows us to observe the effect of different metals in the LnF_3_ compounds. The results agree with the previous set of experimental and computational characterization performed to obtain the real surface image of these systems.^21,22^ We show that EGA-MS allows us to identify all the ions attached to the NP surface and to know the coordination of the multidentate ligand (*e.g.* citrate), disclosing if it is adsorbed by one or two carboxylate moieties.

## Experimental section

### Materials

Yttrium(iii) acetate hydrate 99.9%, lanthanum(iii) acetate hydrate 99.9%, citric acid 99%, tetramethylammonium hydroxide 25% v/v in water and ammonium fluoride > 99.99% were purchased from Sigma-Aldrich. Ethanol 96% was purchased from Panreac and acetone 99.5% from Scharlau. All reagents were used as received without further purification.

### Nanoparticle synthesis

In a 50 mL round-bottom flask equipped with a condenser and a magnetic stirrer, citric acid (2.25 mmol) in 16 mL of MilliQ water was neutralized with tetramethylammonium hydroxide (6.75 mmol), followed by the addition of Ln(CH_3_COO)_3_·H_2_O (1.5 mmol). The initial solution was heated until 100 °C or cooled to 5 °C, and then NH_4_F (4.5 mmol) in 4 mL of MilliQ water was injected dropwise. After 2 h of reaction, the final mixture was cooled to room temperature. LnF_3_ particles were separated from the reaction medium by the addition of 10 mL of ethanol (supraparticles) or acetone (nanoparticles), followed by centrifugation at 10 000 rpm for 20 minutes. The separated nanoparticles were re-dispersed in 20 mL of MilliQ water forming a stable dispersion.

### Characterization

Dynamic light scattering (DLS) analyses have been carried out at the Characterization of Soft-Materials Services at ICMAB using a Zetasizer Nano Zs with a measurement range of 0.3 nm to 10.0 μm and a sensitivity of 0.1 mg mL^−1^. Transmission electron microscopy (TEM) micrographs were obtained on a 120 kV JEOL 1210 TEM, which has a resolution point of 3.2 Å. Nanoparticles were washed five times with a non-solvent (ethanol or acetone), re-dispersed in water and dried with a N_2_ current. The EGA-MS setup consists of a quartz tube that is kept at 10^−6^ mbar. To measure the sample temperature, samples are placed directly on a platinum sheet that is in tight thermal contact with a K thermocouple. Gases evolving from the sample are analyzed by means of a quadrupole mass spectrometer, MS, (model Microvision Plus from MKS). The energy of ionizing electrons is 70 eV. The MS is placed in a chamber next to the quartz tube so that the distance from the sample to the gas analyzer is about 40 cm. The quartz tube is placed inside a tube furnace. Experiments are performed at a constant temperature rise of 5 K min^−1^ or 10 K min^−1^. This setup allows us to monitor the evolution of the volatiles and fragments as a function of the sample temperature.

## Results and discussion

### Nanoparticles

**Fig. 1:**
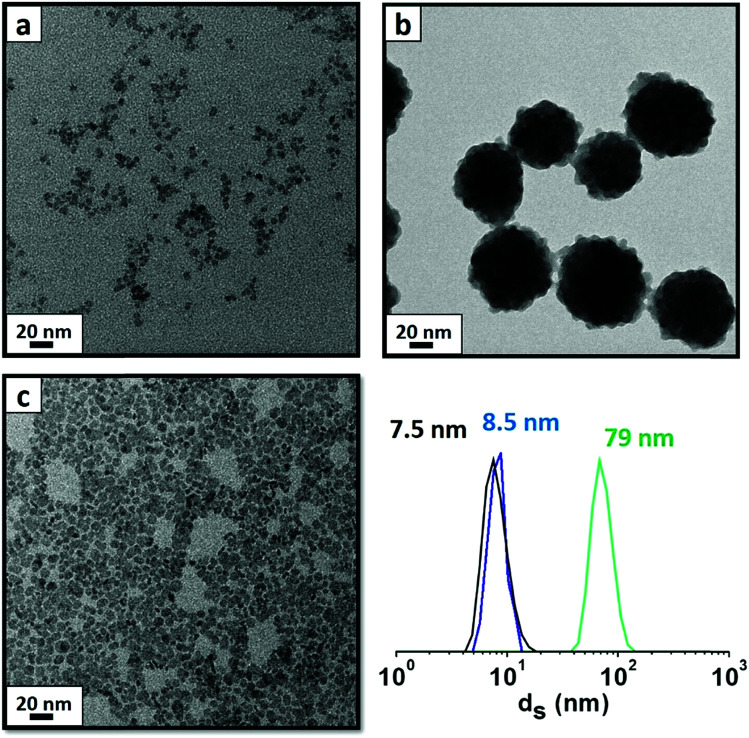
DLS and TEM brightness images of the selected particles for the EGA-MS study. (a) YF_3_ nanoparticles synthesized at 5 °C (DLS in black), (b) YF_3_ supraparticles obtained at 100 °C (DLS in green) and (c) LaF_3_ nanoparticles obtained at 100 °C (DLS in blue), all of them *via* a co-precipitation method in water.

Particles were obtained following a previous study,^23^ based on a modified co-precipitation method in water at 100 °C to obtain YF_3_ supraparticles and LaF_3_ NPs. To obtain YF_3_ NPs with a small size (∼5 nm), we used kinetic control during the synthesis at a temperature of 5 °C.^21^ As shown in Fig. 1, these NPs are monodispersed in water and show a cubic crystalline structure (YF_3_ NPs and supraparticles)^21^ and a hexagonal crystalline structure for LaF_3_ NPs.^22^ During the synthesis, several ionic species were introduced: acetate (from the metallic precursor), tetramethylammonium citrate (stabilizer) and ammonium (counterion of the fluorinating source). Previous studies^21^ have shown the use of different cations in these systems to have an influence, not only in the NP size but, in general trends, on the behavior and charge density.^24,25^ The final composition of the system in this study contains two carboxylate species and two nitrogen-based compounds, impeding direct identification of all adsorbed species by classical experimental techniques.

In previous studies,^21,22^ we used a set of experimental techniques in combination with MD simulations to identify all species present on the surface and to create the complete image of the system. Several techniques were necessary due to the complexity of studying the surface chemistry of these particles. From MD simulations we found that the surface of YF_3_ particles and LaF_3_ particles is spontaneously covered by acetate, citrate and ammonium ions, while tetramethylammonium plays a counterion role neutralizing the global negative charge of the particles (see Fig. S1 in the ESI†).^21,22^

Given the drawbacks of commonly used experimental techniques, the implementation of a general protocol to characterize the surface of nanoparticles is required. Besides, EGA-MS is a suitable technique to detect the volatiles released from the NP surface during thermal treatment. The high sensitivity and reliability of MS make it an advantageous technique to be applied in nanoscale systems containing inorganic NPs. Compared to the traditional measurement based on a TG coupled to EGA, the use of EGA in a vacuum significantly increases the accuracy and prevents the occurrence of secondary reactions or reactions with residual gases that may affect the characterization of the groups evolved from the surface. Also, volatile fragments on their way to the detector are strongly reduced because under the working conditions, the mean free path is much longer than the distance from the sample to the detector (see Fig. S2† as an example of EGA-MS at a determined temperature showing all the volatile fragments identified in this work).

### Monovalent species by EGA-MS

**Fig. 2:**
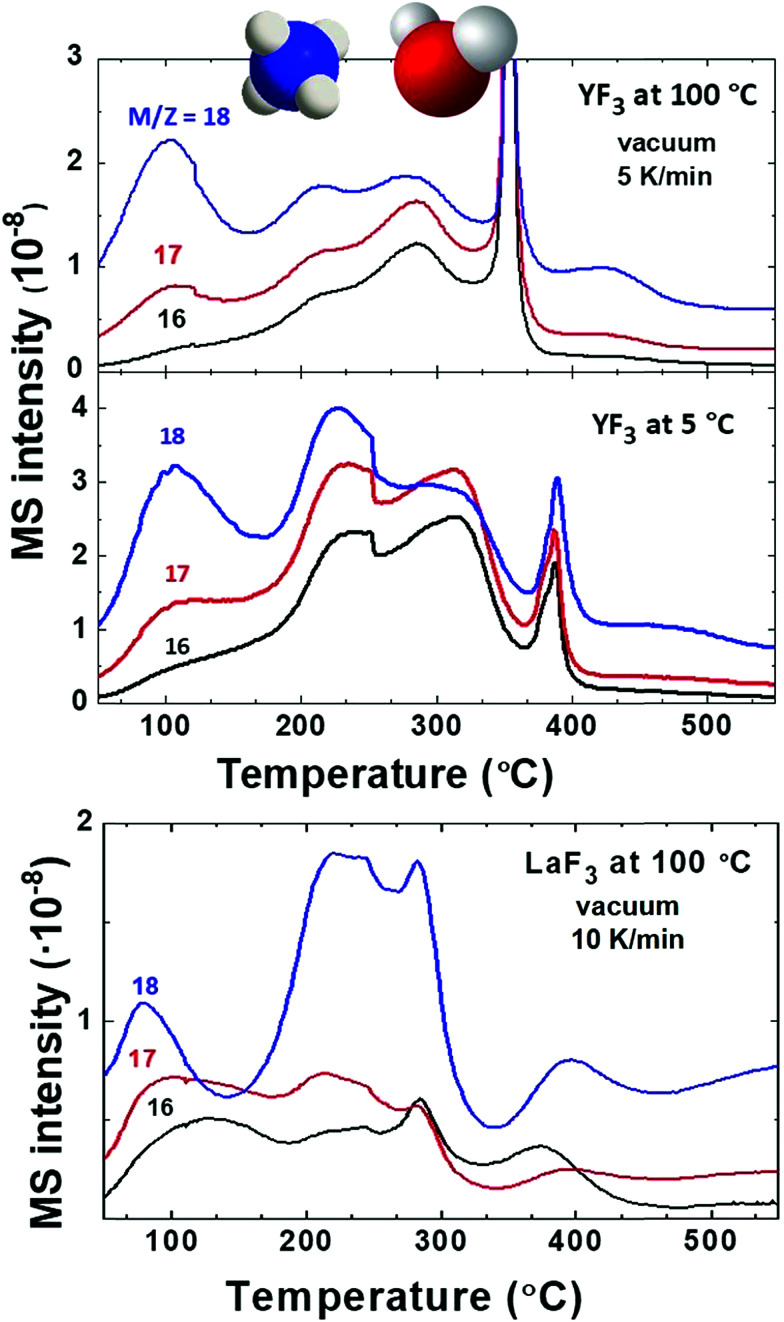
EGA-MS analysis of YF_3_ particles synthesized at 100 °C and those synthesized at 5 °C and LaF_3_ NPs synthesized at 100 °C. All spectra are shown with detected *m*/*z* main fragments of water and ammonia, corresponding to water and ammonium cations adsorbed on the NP surface.

First, we compare YF_3_ NPs (Fig. 1a) to YF_3_ supraparticles (Fig. 1b). EGA-MS allows detection of the ligands attached to the NP surface. Also, the evolution of the fragments as the temperature increases is related to the kinetics of the decomposition and provides information about the stability of the different ligands; we have detected the presence of NH_4_^+^ on the surface of the NPs as well as strongly adsorbed water, playing a stabilizer role. The mass-to-charge ratio (*m*/*z*) fragments of 16, 17 and 18 (Fig. 2) can be interpreted as arising from H_2_O and NH_3_. Their relative intensities should be compared to the fragmentation pattern of H_2_O (I16/I17/I18 = —/0.22/1) and NH_3_ (I16/I17/I18 = 0.8/1/—) as shown in Scheme S1.† The peak at 100 °C corresponds mainly to water desorption with a non-negligible contribution of NH_3_ as revealed by the *m*/*z* = 16 signal. As the temperature increases above 200 °C, two additional processes at around 220 and 300 °C are detected in which the contribution of NH_3_ dominates (higher intensity of 16 and 17 fragments). Finally, the sharp peak between 375 and 400 °C can be attributed to a combustion process. It does not provide any relevant information as most measured *m*/*z* fragments are embedded in this peak. In addition, LaF_3_ NPs show a similar plot with a high adsorbed water contribution of the *m*/*z* fragment 18. EGA-MS has evidenced that water and ammonium are adsorbed onto the NP/supraparticle surface (at the chamber pressure, the boiling point of free water is around −70 °C). These two molecules are hard to detect with the experimental techniques used in previous studies, and XPS and IR were needed to observe the presence of ammonium (see Fig. S1†) because a suitable technique depends on the particular NPs.

**Fig. 3:**
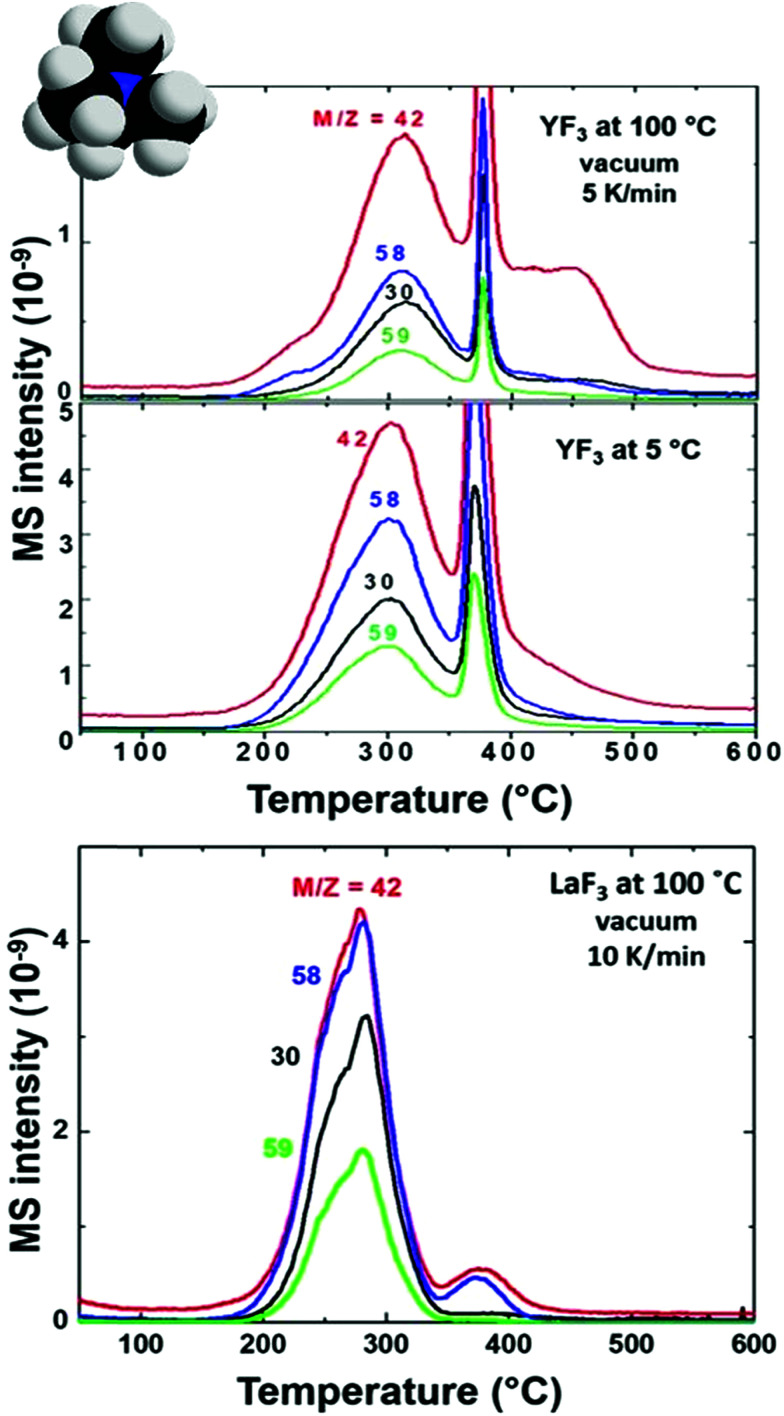
EGA-MS analysis of YF_3_ particles synthesized at 100 °C and 5 °C and LaF_3_ NPs synthesized at 100 °C. All spectra are shown with detected *m*/*z* main fragments of tetramethylammonium release as trimethylamine.

The presence of tetramethylammonium in our system has also been stated as trimethylamine for all NP and supraparticle systems, thanks to the detection of fragments *m*/*z* = 58 and 59 (see Fig. 3) with a ratio of I59/I58 = 0.4 (see the fragmentation path in Scheme S2†). The main peaks for trimethylamine identification (coming from the tetramethylammonium cation) are *m*/*z* = 58, 59, 30 and 42 with expected ratios of (I58/I59/I30/I42 = 1/0.70/0.35/0.27). The exceeding contribution of fragment *m*/*z* = 58 is related to the simultaneous release of acetate to form acetone.^26,27^ Acetone (coming from acetate) can be detected from the presence of *m*/*z* = 58 and 43 with an expected ratio of I43/I58 = 4 as shown in Scheme S3.† Also, the contribution of fragments *m*/*z* = 30 and 42 is larger than expected; the higher amount of small fragments is due to an enhancement of spontaneous dissociation with temperature.^28–30^

**Fig. 4:**
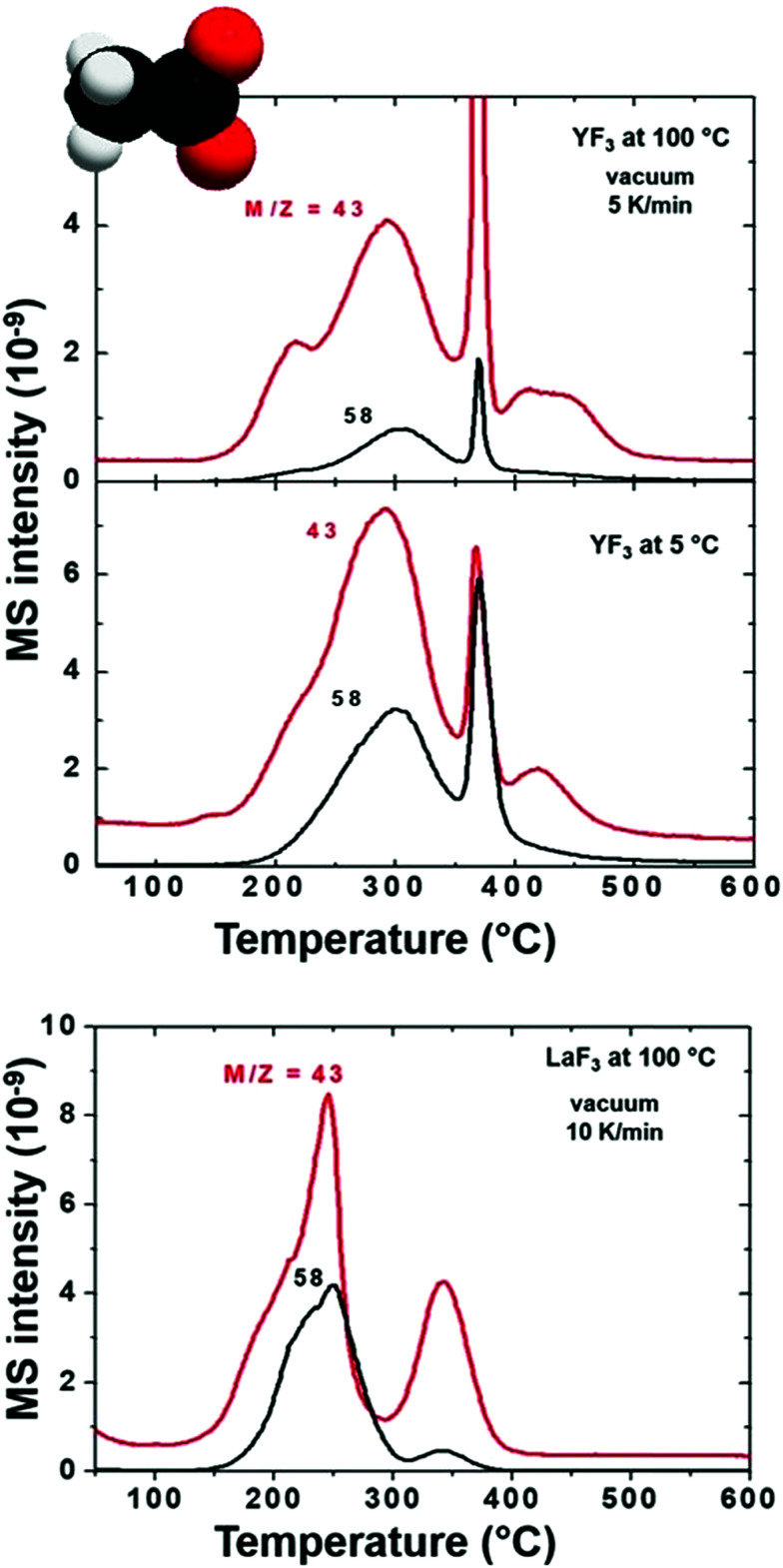
EGA-MS analysis of YF_3_ particles synthesized at 100 °C and 5 °C, as well as LaF_3_ NPs synthesized at 100 °C. All spectra are shown with detected *m*/*z* main fragments of acetate release as acetone.

The presence of acetate on the NP and supraparticle surface (Fig. 4) is confirmed although the relative intensities of the *m*/*z* peaks cannot be determined due to the overlapping with tetramethylammonium fragments. Concerning EGA-MS results of YF_3_ particles *vs.* LaF_3_ NPs, we observe the same volatiles, but with an interesting difference: molecules/ions attached to fluorine atoms (ammonium) on the NP surface are released at the same temperature while the ones attached to lanthanide atoms (acetate and water) evolve at a lower temperature. This effect is related to the different behavior of the metals (*e.g.* their cationic radii).

Tetramethylammonium decomposition plots (Fig. 3) show significant differences in its release temperature, meaning that it is not directly attached to fluorine surface positions. This phenomenon could be explained considering the role of counterions in this system. If tetramethylammonium is attached to fluoride positions, all NPs (LaF_3_ and YF_3_) should have the same behavior. In our case, we observed a lower temperature for LaF_3_ than for YF_3_ (Fig. 3). Tetramethylammonium plays the role of a counterion stabilizing the negative charge coming from citrates. It is released at a different temperature from the NP surface due to the different charge densities of the metals (La^3+^*vs.* Y^3+^) and their citrate–metal interactions, affecting the binding energy between citrate and tetramethylammonium, as explained below for the case of citrate and summarized in Fig. S3.†

### Unravelling the coordination of citrate

**Fig. 5:**
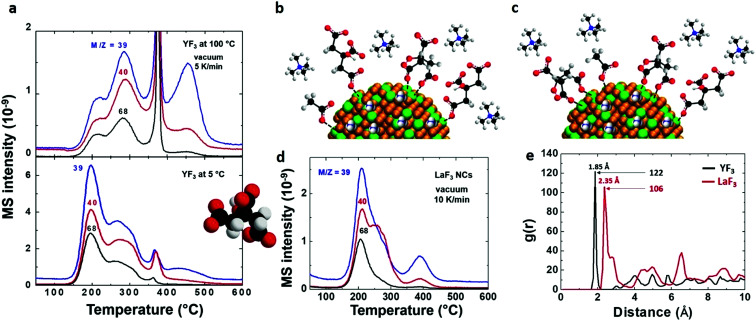
Unravelling the coordination of citrate on the NP surface. (a) EGA-MS analysis of YF_3_ particles synthesized at 100 °C (upper) or 5 °C (bottom) with detected *m*/*z* peaks for citraconic anhydride. Schematic indicating the coordination of different ions on the NP surface for YF_3_ particles synthesized at (b) 5 °C and (c) 100 °C. (d) EGA-MS analysis of LaF_3_ particles with corresponding *m*/*z* peaks of citraconic anhydride. (e) Radial correlation function *g*(*r*) between oxygen atoms of citrate and metal atoms of the NP surface obtained for YF_3_ and LaF_3_ in MD simulations at 100 °C.^21,22^

To detect citrate attached to the NP surface, first we need to know the decomposition path of this molecule. Two decomposition paths for citric acid have been described in the literature:^31^ one at 175 °C with the formation of citraconic anhydride and the other at 250 °C with the formation of 1,3-acetonedicarboxylic acid. Under the conditions used in this study, we expect the formation of citraconic anhydride because it takes place at a lower temperature (Scheme S4†). In consequence, the detection of citraconic anhydride (that has been identified through fragments *m*/*z* = 39, 40 and 68 as shown Fig. 5a) is crucial. The intensity ratios of fragments 39 and 40 agree with the fragmentation pattern of citraconic anhydride (I39/I40/I68 = 1/0.7/0.7). Again, the lower intensity of the fragment *m*/*z* = 68 can be attributed to thermally induced dissociation that favors the formation of smaller fragments.^28–30^

**Scheme 1:**
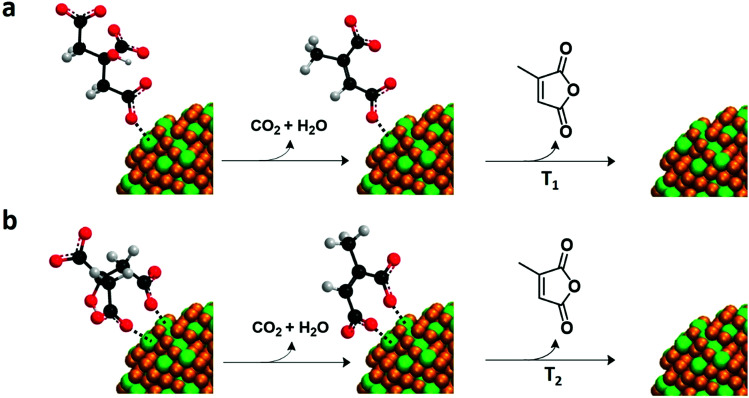
Mechanism involved in the decomposition and release of citrate from the NP surface. (a) Citrate is attached to one carboxylic acid and is released at temperature *T*_1_ and (b) citrate is adsorbed by two different carboxylic moieties and is released at *T*_2_ as citraconic anhydride.

From the EGA-MS curves, we can identify the presence of citraconic anhydride in YF_3_ particles thanks to the peaks of the above fragments at 200 and 285 °C (Fig. 5a). The peak at 200 °C is more intense in particles obtained at 5 °C while the peak at 285 °C is predominant for those synthesized at 100 °C. This difference reveals the dependence of citrate coordination onto the NP surface on the synthesis temperature. At 5 °C, the low thermal energy of the system explains the preferential coordination with one carboxylic group. This interaction could be broken by a mechanism that starts from citrate to form citraconic anhydride, releasing water and carbon dioxide (Scheme 1a). Finally, citraconic anhydride is released from the surface of NPs at a temperature (*T*_1_) of approximately 200 °C. On the other hand, for particles synthesized at 100 °C, the coordination with two different carboxylic moieties accounts for the most intense peak at a higher temperature (*T*_2_ = 285 °C), as can be observed in Scheme 1b.

These two citrate coordinations in YF_3_ NPs and supraparticles were already postulated in our previous work *via*^1^H NMR analysis and MD simulations.^21^ From the present EGA-MS results, we can establish a surface chemistry containing ammonium, acetate, citrate and tetramethylammonium in YF_3_ NPs/supraparticles, *i.e.* the same composition that was identified using several experimental techniques and MD simulations. In addition, given the two different volatilization temperatures for the same molecule, we postulate a surface with two different kinds of coordination for citrate whose relevance depends on the preparation temperature.

Concerning the citrate bridge between YF_3_ NPs postulated as the main reason for their self-assembly into YF_3_ supraparticles,^21^ we consider that the citrate fragments contributing to this bridge evolve at a temperature similar to that for mono- and bi-coordinated citrates and, consequently, their EGA-MS signals are overlapped.

In the case of LaF_3_ particles, EGA-MS gives a single peak related to the three main fragments of citraconic anhydride at 210 °C (Fig. 5d). This means that we have one single kind of citrate coordination onto the NP surface. However, it cannot be directly assigned to a given coordination from only the data of EGA-MS of YF_3_ particles, due to the difference in charge density between Y^3+^ and La^3+^ cations. Our previous MD simulations^22^ predict that each adsorbed citrate moiety is coordinated to the LaF_3_ NP surface by two carboxylic groups. Therefore, the presence of a single coordination mode in MD simulations is consistent with the presence of a single peak in EGA-MS.

The comparison between the results obtained in the case of YF_3_ and LaF_3_ also provides additional physical insight. We observe that water and acetate are released from the NP surface at lower temperatures in LaF_3_ than in YF_3_. This indicates a stronger interaction of these molecules with Y^3+^ which could be due to the smaller radius of yttrium. These differences in the interaction of organic molecules with metals of different sizes are common in coordination complexes in which the charge density of the metal cations is highly influenced by their size. An analogous effect should be expected with citrate. In fact, this is what we obtained in our atomic MD simulations performed for LaF_3_ and YF_3_ in our previous publications.^21,22^ In this case, we used the results of the previous MD simulations to reinforce our affirmation about the different coordination and to clarify this point with the use of this complementary technique.

The results for the citrate–NP interaction are compared for LaF_3_ and YF_3_ in Fig. 5e. In this figure, we show the radial correlation function *g*(*r*) between the oxygen atoms of citrate and NP metal surface atoms obtained in simulations (for detailed discussion on calculation and meaning of the radial correlation function, see ref. 32). The location of the first peak in the *g*(*r*) function indicates the distance between adsorbed citrate oxygen atoms and surface metal ions, and the relative intensity of the peak indicates the strength of the interaction (technically, the potential of mean force). According to Fig. 5e, the oxygen–metal distance is larger for lanthanum (2.35 Å compared with 1.85 Å for yttrium). Furthermore, the first coordination peak is smaller for lanthanum by a factor of ≈0.87 than for yttrium indicating a weaker metal–citrate oxygen interaction for La^3+^. Therefore, MD simulation results imply that citraconic anhydride will be released from the LaF_3_ NP surface at lower temperatures than from YF_3_, due to its weaker interaction (this shift to a lower temperature has also been observed for water and acetate release). This prediction from MD simulations is consistent with the EGA-MS peak temperatures of 285 °C and 210 °C for citraconic anhydride evolution from YF_3_ and LaF_3_, respectively (Fig. 5a and d). Comparing the thermal energies ∼ *K*_B_*T* (*T*, absolute temperature and *K*_B_, Boltzmann constant) associated with these peak temperatures, we obtain a ratio of 4.01 kJ mol^−1^ (corresponding to 285 °C)/4.64 kJ mol^−1^ (corresponding to 210 °C) ≈ 0.86. This value coincides with the ratio between the first peak of the *g*(*r*) function (Fig. 5e) measuring the relative strength of oxygen–citrate–metal interactions for LaF_3_ and YF_3_ NPs discussed above. In summary, we can say that the citrate-related peaks at 285 °C and 210 °C for YF_3_ and LaF_3_, respectively (Fig. 5a and d), correspond to the same decomposition mechanism, *i.e.* that identified at the decomposition temperature *T*_2_ in Scheme 1. The temperatures are different for each metal because of the different strengths of the citrate–metal interaction. Note that EGA-MS and MD simulations are complementary techniques to unravel the dynamics and complex surface chemistry of NPs.

## Conclusions

We report an easy, fast and complete surface characterization method applied to nanoscale systems which allows the identification of all species attached to the NP surface. Using EGA-MS, we unraveled the surface composition of a set of three nanoscale systems (YF_3_ NPs, YF_3_ supraparticles and LaF_3_ NPs). The EGA-MS results are in good agreement with a previous full characterization study that involved several techniques and works as a complementary technique to our previous MD simulations. In other words, EGA-MS is able to provide complete characterization of surface chemistry avoiding the massive use of experimental techniques, but needs ancillary techniques such as MD simulations to assess the complete image.

The surface chemistry of the NPs is composed of a combination of acetate, citrate, ammonium and tetramethylammonium. The same composition is obtained in all cases, but EGA-MS reveals differences in the coordination of the carboxylate moieties of citrate to the metal cation in the NP core. In particular, YF_3_ NPs obtained at 5 °C present a preferential coordination with one carboxylate moiety. On the other hand, YF_3_ supraparticles and LaF_3_ NPs synthesized at 100 °C show a preferential coordination with two carboxylate moieties. The metal present in the NP (Y^3+^ or La^3+^) has a direct influence on the release temperature of the molecules or ions attached to these atoms (water, acetate and citrate) due to a different strength in the metal–molecule or metal–ion interaction (charge density effect). In addition, the counterion role played by tetramethylammonium is also stated thanks to the difference between its decomposition patterns in LaF_3_ and YF_3_ NPs. These differences mean that tetramethylammonium is interacting with citrate adsorbed on the metal cation position, the metal–ligand charge density being the main reason for the difference in thermal stability. We found that the ions attached to lanthanum were released at a lower temperature than those adsorbed on yttrium atoms. This behavior is as expected because of their cationic radii (La^3+^ is bigger than Y^3+^); the bigger the metal radius is the lower the binding energy is. This is also consistent with the NP–ion interaction observed in our previous MD simulations.^21,22^

The present study is of interest not only for metal fluoride NPs but, in general, for all kinds of nanoscale systems in which their surface composition is the key parameter to control and design stable nanoscale suspensions. The use of EGA-MS as one of the main techniques to fully characterize nanoscale systems avoids the necessity of performing several experimental characterization studies to unravel the actual surface picture. Although in some cases the use of different techniques is necessary to gain greater insights into uncovering specific details of the obtained systems (*e.g.* spectroscopic techniques), here we demonstrate the efficiency of EGA-MS in NP surface characterization.

## Conflicts of interest

There are no conflicts to declare.

## Supplementary Material

NA-001-C9NA00098D-s001
