# Supplementary material for: Using evolved gas analysis – mass spectrometry to characterize adsorption on a nanoparticle surface
Source: Nanoscale Adv. 2019 Jun 4;1(7):2740–7. doi: 10.1039/c9na00098d (PMC9417425; doi:10.1039/c9na00098d)
Supplement: NA-001-C9NA00098D-s001 [file NA-001-C9NA00098D-s001.pdf]

Electronic Supplementary Material (ESI) for Nanoscale Advances.
This journal is © The Royal Society of Chemistry 2019

SUPPORTING INFORMATION

Using evolved gas analysis – mass spectrometry to characterize adsorption at a nanoparticle surface

Jordi Martínez-Esaín,[†,‡] Teresa Puig,[‡] Xavier Obradors,[‡] Josep Ros,[†] Jordi Farjas,[§] Pere Roura-Grabulosa,[§] Jordi Faraudo,[‡] Ramón Yáñez[†] and Susagna Ricart[‡,]*

[†] Departament de Química, Universitat Autònoma de Barcelona, Campus de la UAB, 08193, Bellaterra, Catalonia, Spain.

[‡] Institut de Ciència de Materials de Barcelona (ICMAB-CSIC), Campus de la UAB, 08193, Bellaterra, Catalonia, Spain.

[§] University of Girona, Campus Montilivi, Edif. PII, E17071 Girona, Catalonia, Spain

Surface chemistry overview

Previous characterizations to YF_3 and LaF_3 were carried out using a set of experimental techniques and MD simulations to explore the surface chemistry of these nanoparticles (NPs).[1,2] In the case of YF_3 (at both temperatures), we observed that their surface is spontaneously covered by adsorbed ammonium onto fluoride atoms and citrate and acetate onto yttrium atoms while tetramethylammonium were playing a counterion role neutralizing the negative charge of this system (Figure S1a). Experimentally, we found the presence of citrate, acetate and tetramethylammonium via ^{1}H NMR while to detect ammonium was needed the use of XPS analysis (Figure S1d).

Concerning the surface chemistry of YF_3 NPs and supraparticles, we observed that they presented a similar behaviour containing the same stabilizers onto the surface. In fact, via MD simulations we realized that considering the same sized NP (computational performed) at high synthetic temperatures, high amount of stabilizer was attached, but maintaining the relative ratio between them (i.e. the same relative percentage of acetate, citrate and ammonium were observed in both systems).[1]

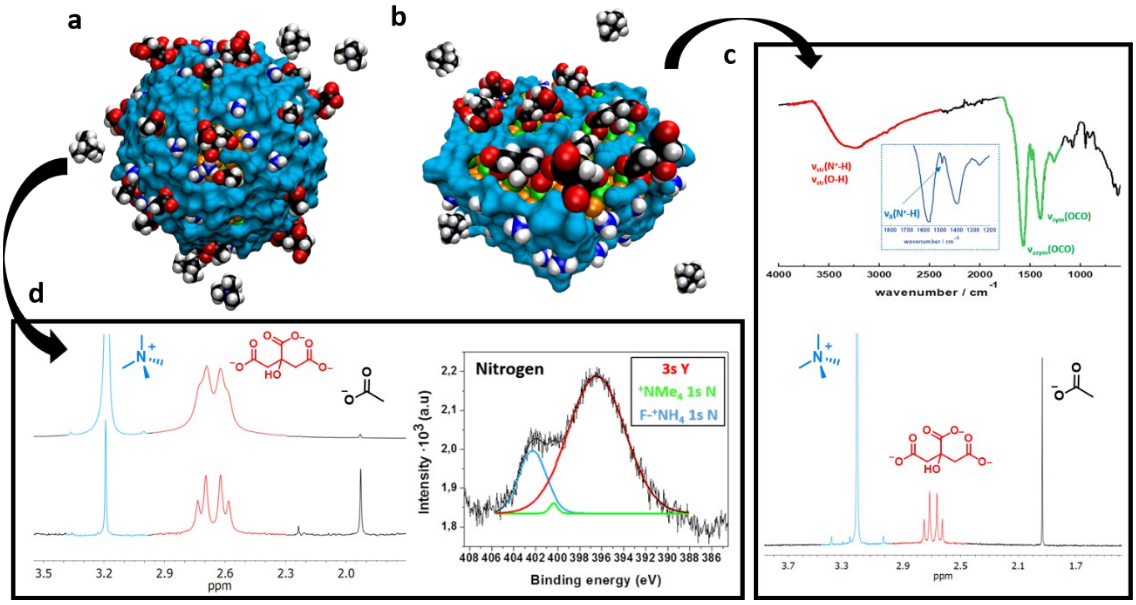

Figure S1. Summary of the experimental characterization and MD simulations performed to unravel the surface image of YF_3 and LaF_3 NPs.[1,2] **(a)** Equilibrium configuration obtained in MD simulation of YF_3 at 100 °C, **(b)** equilibrium configuration obtained in MD simulation of LaF_3 at 100 °C, **(c)** IR and 1H NMR characterization of LaF_3 NPs washed five times and **(d)** ^{1}H NMR (upper YF_3 synthesised at 5 °C and bottom synthesised at 100 °C) and XPS of YF_3 supraparticles synthesized at 100 °C. Concerning MD simulations, MF_3 atoms and adsorbed ligands are shown as spheres with their Van der Waals radius (M green, F orange, C black, O red, N blue, H white). We show adsorbed acetate and citrate ions and adsorbed ammonium ions, while tetramethylammonium cations are playing the counterion role. Water molecules adsorbed to the NC surface are shown in blue as a molecular contour surface (calculated using the surface algorithm implemented in VMD).

In the case of LaF_3 NPs, we observed a selective adsorption of cations and anions in their different facets, forming the as known faceted-charge patchy NPs.[2] We have realized that ammonium cations were adsorbed onto rectangular facets while citrate and acetate were selectively adsorbed onto hexagonal facets. Although the selective distribution could not be directly detected experimentally, MD simulations clearly showed this organisation (Figure S1b). Considering the experimental techniques used to unravel this surface chemistry, in this case we used ^{1}H NMR to detect citrate, acetate and tetramethylammonium and IR to see the bending mode of ammonium cation (Figure S1c).

In our case, the use of a single technique was not enough to clarify the surface chemistry of these NPs. Although, several nanoscaled systems are fully characterized using NMR analysis due to their attached organic molecules,[3–7] in our particular case we need to find other technique, using EGA-MS, to allow the complete detection of all species.

Decomposition patterns

Scheme S1. Expected fragments produced from water and ammonia after its dissociation in the ionization chamber of a mass spectrometer

$$H_2O \longrightarrow \underset{M/Z = 17}{OH^+} + \underset{M/Z = 18}{H_2O^+}$$

$$NH_4^+ \longrightarrow \underset{M/Z = 16}{NH_2^+} + \underset{M/Z = 17}{NH_3^{+\bullet}}$$

Scheme S2. Expected fragments produced by trimethylamine after its dissociation from tetramethylammonium cations.

$$C_4H_{12}N^+ \longrightarrow \underset{M/Z = 30}{CH_2NH_2^+} + \underset{M/Z = 42}{C_2H_4N^+} + \underset{M/Z = 58}{C_3H_8N^+} + \underset{M/Z = 59}{C_3H_9N^+}$$

Scheme S3. Expected fragments produced by acetone after its dissociation from acetate anion.

$$C_2H_3O_2^- \longrightarrow \underset{M/Z = 43}{C_2H_3O^+} + \underset{M/Z = 58}{C_3H_6O^{+\bullet}}$$

Scheme S4. Expected fragments produced by citraconic anhydride acid after its dissociation from citrate.

$$C_6H_5O_7^{3-} \longrightarrow C_3H_3^{+} + C_3H_4^{+\bullet} + C_4H_4O^{+\bullet}$$

M/Z = 39 **M/Z = 40** **M/Z = 68**

Example of M/Z of a typical EGA-MS diagram for a determined temperature

The typical M/Z plot for EGA-MS is shown in Figure S2 chosen YF_3 NPs synthesized at 5 ˚C, measured at 300 ˚C as example. We chose 300 ˚C because in the case of YF_3 NPs synthesized at 5 ˚C all volatiles have been observed in EGA-MS plots showed in the main paper.

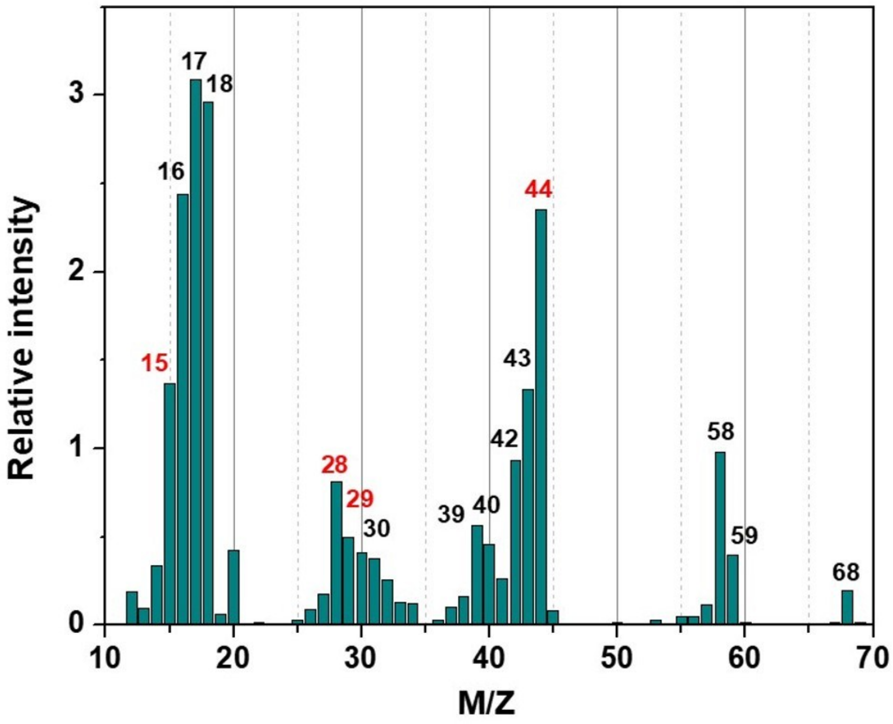

Figure S2. Representative M/Z spectrum for a typical EGA-MS analysis of YF_3 NPs synthesized at 5 ˚C at a measured temperature of 300 ˚C in EGA-MS plots. In black it is shown the main peaks of all volatiles detected during the work corresponding to the assignation of water, ammonium, acetate, tetramethylammonium and citrate compounds. In red are assigned the main secondary volatiles.

All M/Z fragments in black are described in the main paper and summarized in the decomposition patterns in Supporting Information. Concerning the red M/Z fragments, they could be assigned to secondary common volatiles in this kind of processes. M/Z= 15 is assigned to methyl fragmentation, M/Z= 28 is assigned to carbon monoxide, M/Z= 29 is commonly assigned to aldehyde or ethyl fragments and M/Z= 44 corresponds to carbon dioxide.

Ammonium vs tetramethylammonium role

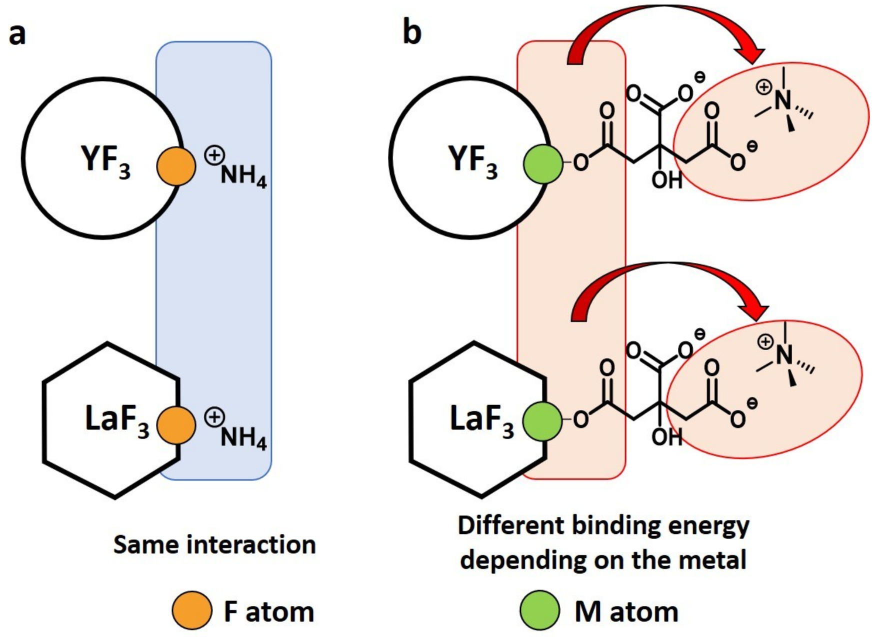

Figure S3. Schematic representation of the cationic interactions in the nanoscaled systems. **(a)** Ammonium cation directly adsorbed onto F atoms show the same binding energy in all systems because there is not direct interaction with metal atom. **(b)** Tetramethylammonium cations playing the counterion role are postulated to interact with citrate adsorbed anions. As the binding energy between metal atom onto the NP surface and citrate is different depending on the metal, we postulated some difference between the binding energies citrate-tetramethylammonium.

Reference

1 J. Martinez-Esain, J. Faraudo, T. Puig, X. Obradors, J. Ros, S. Ricart and R. Yáñez, *J. Am. Chem. Soc.*, 2018, **140**, 2127–2134.

2 J. Martínez-Esaín, T. Puig, X. Obradors, J. Ros, R. Yáñez, J. Faraudo and S. Ricart, *Angew. Chemie - Int. Ed.*, 2018, **5**, 14747–14751.

3 L. E. Marbella and J. E. Millstone, *Chem. Mater.*, 2015, **27**, 2721–2739.

4 T. K. Egner, P. Naik, N. C. Nelson, I. I. Slowing and V. Venditti, *Angew. Chemie - Int. Ed.*, 2017, **56**, 9802–9806.

5 R. R. Knauf, J. C. Lennox and J. L. Dempsey, *Chem. Mater.*, 2016, **28**, 4762–4770.

6 Z. Hens and J. C. Martins, *Chem. Mater.*, 2013, **25**, 1211–1221.

7 J. De Roo, K. De Keukeleere, Z. Hens and I. Van Driessche, *Dalt. Trans.*, 2016, **45**, 13277–13283.
